# PubChem: atom environments for molecule standardization

**DOI:** 10.1186/1758-2946-5-S1-P38

**Published:** 2013-03-22

**Authors:** Volker Hähnke, Evan E Bolton, Stephen H Bryant

**Affiliations:** 1 National Center for Biotechnology Information, National Library of Medicine, National Institutes of Health, Department of Health and Human Services, Bethesda, Maryland, 20894, USA

## 

PubChem is an open repository for molecular structures, their properties and biological activities [1]. The number of deposited structures has been steadily increasing since its creation in 2004. Today, it contains more than 92 million substances (PubChem Substance) with 32 million unique small molecules (PubChem Compound). Consequently, visual inspection of every structure and correction of errors by hand to detect structure equivalencies and to ensure data quality are not feasible. Efficient and reliable automated methods for standardization are necessary during the registration process to compensate for alternating representations of as well as errors and artifacts in (sub)structure representations caused by diverging business rules, personal preferences, data format conversion, disagreements between aromaticity definitions and automated library generation. At PubChem, we are developing a new standardization approach that is based on rules for atom environment transformation. Those rules are obtained from a statistical analysis of atom environment transformations observed with a reference workflow combining chemical reasonability checks, valence filters, canonical tautomer determination and aromaticity normalization. Additional atom environment mappings are provided by hand curation. In the first application of our technique to PubChem we concentrate on purely organic compounds. Those represent 97% of the deposited structures and account for the majority of atom environments as well. Here, we present the first results obtained with our approach, highlighting the methodology, challenges, benefits and future possibilities.
